# Dynamic PET imaging in patients with unilateral carotid occlusion shows lateralized cerebral hypoperfusion, but no amyloid binding

**DOI:** 10.1177/13872877251329593

**Published:** 2025-04-17

**Authors:** Naomi LP Starmans, Anna E Leeuwis, Edwin Bennink, Sebastiaan L Meyer Viol, Sandeep SV Golla, Jan Willem Dankbaar, Esther E Bron, Geert Jan Biessels, L Jaap Kappelle, Wiesje M van der Flier, Nelleke Tolboom, on behalf of the Heart-Brain Connection Consortium

**Affiliations:** 1 Department of Neurology and Neurosurgery, University Medical Center Utrecht, Utrecht, The Netherlands; 2 Alzheimer Center Amsterdam, Department of Neurology, Amsterdam Neuroscience, Amsterdam UMC, location VUmc, Amsterdam, The Netherlands; 3 Department of Medical Psychology, Amsterdam UMC, location VUmc, Amsterdam, The Netherlands; 4 Department of Radiology and Nuclear Medicine, University Medical Center Utrecht, Utrecht, The Netherlands; 5 Department of Radiology and Nuclear Medicine, Amsterdam Neuroscience, Amsterdam UMC, location VUmc, Amsterdam, The Netherlands; 6 Department of Radiology & Nuclear Medicine, Erasmus MC, Rotterdam, The Netherlands; 7 Department of Epidemiology, Amsterdam UMC, Vrije Universiteit Amsterdam, Amsterdam, The Netherlands

**Keywords:** Alzheimer's disease, amyloid-β, carotid artery diseases, cerebral perfusion, dementia, positron emission tomography

## Abstract

**Background:**

Carotid occlusive disease is a risk factor for cognitive decline. A possible underlying etiology is that hemodynamic impairment results in decreased cerebral perfusion, exacerbated amyloid-β accumulation (Aβ) and poorer cognitive performance.

**Objective:**

We aimed to determine whether patients with unilateral internal carotid artery (ICA) occlusion have less cerebral perfusion and more Aβ in the ipsilateral than in the contralateral hemisphere, and whether perfusion and Aβ are associated with cognitive functioning.

**Methods:**

We included 20 patients (age 67.2 ± 7.0 years, 8 females, MMSE 29 [27–29]) with unilateral ICA occlusion, which underwent neuropsychological assessment and dynamic ^18^F-Florbetaben positron emission tomography (PET). Global and regional relative perfusion (R_1_) and binding potential (BP_ND_) were obtained from the PET-images using a simplified reference tissue model. We performed Wilcoxon signed-rank tests to examine differences between hemispheres within subjects and linear regression to investigate associations with cognitive functioning.

**Results:**

Median global R_1_ was 0.911 (0.883–0.950) and global BP_ND_ was 0.172 (0.129–0.187). R_1_ was lower in the hemisphere ipsilateral to the ICA occlusion than in the contralateral hemisphere (0.899 [0.876–0.921] versus 0.935 [0.889–0.970]). BP_ND_ did not differ significantly between hemispheres (ipsilateral 0.172 [0.124–0.181] versus contralateral 0.168 [0.137–0.191]). Neither cerebral perfusion nor Aβ burden were associated with cognitive functioning.

**Conclusions:**

Patients with unilateral ICA occlusion did not have more Aβ in the ipsilateral hemisphere than in the contralateral hemisphere despite ipsilateral hypoperfusion. Perfusion and Aβ were unrelated to cognitive functioning. This indicates that cognitive impairment in patients with ICA occlusion is not due to exacerbated Aβ accumulation.

## Introduction

Carotid occlusive disease (COD) is a risk factor for cognitive decline.^1^ One potential etiology is impaired cerebral hemodynamics. In patients with COD, reduction in cerebral perfusion is initially countered by vasodilation.^2,3^ With increasing hemodynamic compromise, when this compensation mechanism is exhausted, actual cerebral perfusion drops and oxygen extraction fraction increases.^2,3^ Patients with a unilateral internal carotid artery (ICA) occlusion provide the rare opportunity to study hemodynamic impairment in vivo within patients, in which the hemisphere ipsilateral to the ICA occlusion is expected to be more severely impacted than the contralateral hemisphere. Some previous studies have indeed found a lower cerebral perfusion in the ipsilateral hemisphere.^4–7^

An important outstanding question is whether the reduced cerebral perfusion results in exacerbated deposition of amyloid-β (Aβ), as this could provide a potential treatment target for cognitive impairment in COD.^8^ Earlier studies in patients with unilateral ICA stenosis or occlusion did not report a higher cerebral Aβ burden in the ipsilateral hemisphere than the contralateral hemisphere.^7,9–13^ However, patient numbers in these studies were small, and only one small study investigated both cerebral perfusion and Aβ burden in the same cohort.^7^ Furthermore, the positron emission tomography (PET) scans were mostly static, which might underestimate the Aβ burden.

Using dynamic PET instead of static PET could overcome those issues. Dynamic PET can be used for quantitative assessment of both cerebral perfusion and cerebral Aβ burden, allowing to estimate both brain pathologies at the same time. Moreover, dynamic PET enables full quantification of the Aβ load by correcting for individual differences in cerebral perfusion.^14^ These individual differences in cerebral perfusion may result in differences in radiotracer delivery to and clearance from the brain, which can result in spurious results for estimation of Aβ binding, when using semi-quantitative measures like the standardized uptake value ratio.^14^ This correction is especially relevant in patients with ICA occlusion with a potentially reduced cerebral perfusion.

In this study, we intended to investigate the effects of hemodynamic impairment in a specifically selected group of patients with a unilateral, complete occlusion of the ICA. We aimed to determine with dynamic PET if these patients have less perfusion, accompanied by more Aβ, in the hemisphere ipsilateral to the occlusion than in the contralateral hemisphere. Additionally, we examined whether the global cerebral perfusion and Aβ burden are associated with cognitive functioning.

## Methods

### Study population

We conducted a prospective observational study in the University Medical Center Utrecht, The Netherlands, between 2021 and 2023, as a part of the Heart-Brain Connection Study.^15^ For the current study, new data was acquired. The main inclusion criterion of this new study was a unilateral occlusion of the ICA visible on magnetic resonance angiography (N = 8), computed tomography angiography (N = 10) or digital subtraction angiography (N = 2). Additionally, patients had to be 55 years or older and be able to undergo the PET-scan procedures. They had to have a Mini-Mental State Examination (MMSE) score of 18 or higher, thereby allowing participants with cognitive impairment to enter the study, but excluding participants that are too cognitively impaired to give informed consent or to adequately follow instructions during the dynamic PET-scan. We strived for as little stenosis as possible in the contralateral ICA and middle cerebral artery (MCA; contralateral ICA or MCA stenosis of less than 50% in all patients). Patients were also excluded if they had a history of vascular reconstructive surgery in the neck or brain, had structural abnormalities on brain magnetic resonance imaging (MRI) that were likely to interfere with the clinical presentation or interpretation of the PET-scan, participated in an experimental study with an Aβ targeting agent or had a history of another neurological or major psychiatric disorder.

We included 21 patients and scheduled them for a structured interview, brain MRI-scan and dynamic Aβ PET-scan within a period of two months. One patient dropped out of the study prematurely, because the PET-scan had to be rescheduled last-minute due to a failed production of the radiotracer. This patient was excluded from the present analyses, leaving us with the intended sample size of 20 patients. Based on the repeated measures design comparing the ipsilateral to the contralateral hemisphere, a presumed alpha of 0.05 and a power of 0.80, an effect size of dz 0.58 can be demonstrated in this sample of 20 participants.

### Ethics statement

The study has been approved by the institutional review board of the University Medical Center Utrecht, The Netherlands, and was conducted in accordance with the Declaration of Helsinki. All subjects signed an informed consent form.

### Clinical information

Demographic information, medical history and medication use were recorded during a structured interview. Hyperlipidemia was defined as a self-reported history of hyperlipidemia. Diabetes mellitus was defined as a self-reported history of diabetes mellitus or the use of anti-diabetic medication. History of transient ischemic attack or ischemic stroke was based on a self-reported history of those events in the hemisphere ipsilateral to the occlusion. Known duration of the occlusion was defined as the time from the first detection of the occlusion on any imaging method until the participation in the study. We measured height, weight and resting blood pressure.

### Neuropsychological assessment

Participants underwent a standardized neuropsychological assessment with tests for global cognitive functioning and four major cognitive domains, namely memory, language, attention and psychomotor speed and executive functioning.^15^ We calculated z-scores from the raw test scores of the individual tests with the healthy, control participants from the Heart-Brain Connection Study as the reference group.^15^ The z-score for each cognitive domain (secondary outcome) was then calculated as the average z-scores in that domain. Global cognitive functioning (main outcome) was calculated as an average z-score across the four cognitive domains. A cognitive domain was rated as impaired if the z-score was ≤ −1.5.

We used the MMSE and Montreal Cognitive Assessment (MoCA) as cognitive screening tests.

### Magnetic resonance imaging scan acquisition and processing

We acquired a 3 T brain MRI on a Philips Ingenia scanner (Philips, Best, The Netherlands), which encompassed T1-weighted images (resolution 1 × 1 × 1 mm^3^, magnetization-prepared rapid acquisition gradient echo, repetition time 8.2 ms, echo time 4.5 ms, shot interval 3000 ms, flip angle 8°, inversion delay 990 ms) and fluid-attenuated inversion recovery images (FLAIR; resolution 1.11 × 1.11 × 1.11 mm^3^, repetition time 4800 ms, echo time 313 ms, inversion time 1650 ms, turbo spin-echo factor 182).^15^

We applied a brain tissue and white matter hyperintensity (WMH) segmentation method to the T1-weighted images and FLAIR images (Quantib BV, Rotterdam, The Netherlands).^16^ First, the intracranial volume was determined. We then manually segmented infarcts and measured the total brain volume and WMH volume excluding these infarcts regions. We calculated the whole brain parenchymal fraction as the total brain volume divided by the intracranial volume. We expressed WMH volume as the percentage of the intracranial volume.

### Positron emission tomography scan acquisition and processing

Patients underwent a dynamic positron emission tomography-computed tomography (PET-CT) scan on a clinical PET-CT scanner (Siemens Biograph 40 mCT, Siemens, Munich, Germany) with ^18^F-Florbetaben as the radiotracer (NeuraCeq^®^; Curium, Paris, France), which has highly selective binding for Aβ. We used a 60-min dual-time-window acquisition protocol.^17^ A low dose CT-scan (effective 120 kV, 30 mAs) was first acquired for attenuation correction. Patients then received an intravenous bolus of 273 ± 61 MBq ^18^F-Florbetaben, after which the first PET-scanning session of 30 min (6 × 5, 3 × 10, 4 × 60, 2 × 150, 2 × 300 and 1 × 600 s) was immediately initiated. This was followed by a 60-min break, a second low dose CT-scan (effective 120 kV, 30 mAs) for attenuation correction and finally the second PET-scanning session of 20 min (4 × 5 min). PET-images were reconstructed with the TrueX algorithm, an iterative reconstruction with 4 iterations and 21 subsets, including time-of-flight, point spread function modeling and a 5 mm Gaussian filter.

PET-scans were analyzed in PMOD 4.205 with the PNEURO add-on (PMOD Technologies Ltd, Zürich, Switzerland). Both PET-scans were combined into a single multi-frame PET-image, with all time-frames co-registered. T1-weighted images were then also co-registered to the combined PET-image. We measured the cortical relative perfusion (R_1_) and binding potential relative to the non-displaceable compartment (BP_ND_) using a simplified reference tissue model with the entire cerebellar gray matter as the reference region.^18–20^ The transformation from MNI atlas space to the T1-weighted images was automatically calculated in PMOD, after which the Hammers N30R83.^21,22^ and watershed atlases^23,24^ were deformed to match the image. The infarct regions assessed at the MRI-scans were re-used for the PET-scans. For the PET-analyses only, we mirrored the infarct regions onto the contralateral hemisphere, as comparing a hemisphere with an infarct to a hemisphere without an infarct would provide unreliable results. There is namely no Aβ accumulation in the infarct regions, but it is possible for Aβ to accumulate in the contralateral cortex without infarct. By excluding the infarct regions on both sides, the volume of tissue at risk for Aβ accumulation is equal again, thereby reducing the risk of false differences in Aβ burden between the hemispheres. Both the mean R_1_ and the mean BP_ND_ for the Hammers N30R83 atlas regions and watershed areas were determined excluding these infarcts regions on both sides.

Global R_1_ and BP_ND_ covered the entire cerebral cortex and were measured for characterization of our cohort and for assessing associations with cognitive functioning. We checked for crossed cerebellar diaschisis by comparing the R_1_ of the ipsilateral cerebellar cortex to the R_1_ of the contralateral cerebellar cortex. Since the entire cerebellar gray matter is selected as reference region, any interhemispheric differences in the cerebellum are preserved. We examined the R_1_ and BP_ND_ in the entire hemisphere as main outcome for the within patient analyses. In addition to the hemispheric R_1_ and BP_ND_, we assessed 1) the individual lobes (i.e., frontal, temporal, parietal and occipital lobe), 2) the regions that are the first to become amyloid-positive in Alzheimer's disease (i.e., anterior cingulate, posterior cingulate, paracentral gyrus, lateral orbitofrontal cortex, medial orbitofrontal cortex, precuneus and insula)^25^ and 3) the regions that are most at risk for cerebral hypoperfusion (i.e., the watershed areas between the flow territory of the anterior cerebral artery and MCA as well as between the MCA and posterior cerebral artery)^23,24^ as secondary outcomes for the within patient analyses. Next, a R_1_-delta and BP_ND_-delta were calculated as, respectively, the R_1_ or BP_ND_ of the ipsilateral hemisphere minus the R_1_ or BP_ND_ of the contralateral hemisphere. Patients with a R_1_-delta of less than 0 were classified as having ipsilateral hypoperfusion. Patients with a BP_ND_-delta of more than 0 were defined to have an increased ipsilateral cerebral Aβ burden.

Apart from the quantitative analyses, an experienced nuclear medicine physician visually (NT) rated the PET-images according to the manufacturer's instructions to determine amyloid positivity.

### Statistical analyses

Data distribution was assessed by visual inspection of histograms and Q-Q plots. WMH volumes were log transformed to achieve a normal data distribution. No other variables were transformed.

We first evaluated the differences in R_1_ between the cerebellum, hemispheres, lobes, early Alzheimer's regions and watershed areas ipsilateral and contralateral to the ICA occlusion with Wilcoxon signed-rank tests. We then determined the differences in BP_ND_ in the same regions, except for the cerebellum. As sensitivity analyses, we repeated the analyses for BP_ND_ restricting the analyses to patients with ipsilateral hypoperfusion.

Next, we assessed the association between cerebral perfusion and cerebral Aβ burden, both within hemispheres and between hemispheres with the R_1_-delta and BP_ND_-delta. We calculated both crude coefficients and coefficients adjusted for age and sex with multiple linear regression. Additional adjustment for covariates such as vascular risk factors and COD characteristics was not possible given the limited sample size of our cohort. We also assessed whether age, sex, known duration of the occlusion, WMH volume and global R_1_ would affect the interhemispheric differences in R_1_ by assessing their association with the R_1_-delta, again calculating crude coefficients as well as age- and sex-adjusted coefficients. We then determined similar associations for the BP_ND_-delta.

Finally, we determined the associations between global R_1_ or BP_ND_, global cognitive functioning and the specific cognitive domains. For these analyses specifically, global R_1_ or BP_ND_ were standardized to facilitate comparison. We used multiple linear regression to calculate both crude coefficients and coefficients adjusted for age, sex and years of education. Additional adjustment for other potentially relevant covariates was not possible due to the limited sample size. We performed several sensitivity analyses: 1) repeating the analyses for BP_ND_ without the only visually amyloid-positive participant, 2) determining associations with lobar instead of global R_1_ or BP_ND_ and 3) testing for possible interactions between age, sex, R_1_ and BP_ND_ by adding an interaction term to the models.

We applied false discovery rate (FDR) correction to correct for multiple testing. Statistical significance was defined as a p-value < 0.05 after FDR-correction. Analyses were performed in R version 4.0.3.

## Results

Patients had a mean age of 67.2 ± 7.0 years and 8 (40%) were female (Table 1). Eight patients had an occlusion of the left ICA and 12 of the right ICA. The median known duration of the occlusion was 7.1 years (interquartile range (IQR) 0.9–12.4). Median scores for MMSE and MoCA were 29 and 26, respectively.

**Table 1.** Demographics of the patients.

Characteristics	Entire cohort N = 20
Female	8 (40)
Age, y	67.2 ± 7.0
Education level of Verhage ≥ 5*	16 (80)
*Vascular risk factors*	
Current smoking	5 (25)
Systolic blood pressure, mmHg	147.5 ± 20.9
Diastolic blood pressure, mmHg	79.1 ± 15.1
Hyperlipidemia	13 (65)
Diabetes mellitus	5 (25)
Body mass index, kg/m^2^	28.5 ± 3.2
History of TIA or ischemic stroke ipsilateral to ICA occlusion	18 (90)
*Medication*	
Anti-hypertensive medication	16 (80)
Lipid lowering medication	16 (80)
*COD characteristics*	
Left sided occlusion	8 (40)
Known duration of the occlusion, y	7.1 (0.9–12.4)
Contralateral ICA stenosis	
- 0–30%	16 (80)
- 31–50%	4 (20)
*Cognitive functioning*	
MMSE, score	29 (27–29)
MoCA, score	26 (23–27)
Global cognitive functioning, z-score	−0.29 (−0.99 – −0.11)
Memory domain, z-score	−0.07 (−0.91–0.48)
Language domain, z-score	−0.53 (−0.86 – −0.28)
Attention-psychomotor speed domain, z-score	−0.73 (−1.98 – −0.23)
Executive functioning domain, z-score	−0.24 (−0.62–0.13)
*Amyloid-β burden*	
Global BP_ND_	0.172 (0.129–0.187)
*Atrophy and cerebral vascular disease burden*	
Brain parenchymal fraction, ratio	0.78 ± 0.03
Global cerebral perfusion, R_1_	0.911 (0.883–0.950)
WMH volume, % of intracranial volume	0.09 (0.04–0.26)

Data are presented as number (percentage) for categorical variables and mean ± standard deviation or median (interquartile range) for continuous variables.

BP_ND_: binding potential; COD: carotid occlusive disease; ICA: internal carotid artery; MMSE: Mini-Mental State Examination; MoCA: Montreal Cognitive Assessment, R_1_: relative perfusion; TIA: transient ischemic attack; WMH: white matter hyperintensities.

### Cerebral perfusion

The median global R_1_ was 0.911 (IQR 0.883–0.950). Cerebellar R_1_ was similar on the ipsilateral and contralateral side (R_1_ ipsilateral 1.004 [IQR 0.996–1.012] versus contralateral 0.997 [IQR 0.988–1.005], p = 0.430). Seventeen of 20 patients had a R_1_-delta of less than 0, i.e., ipsilateral hypoperfusion (median R_1_-delta of −0.024 [IQR −0.045 – −0.009]). The remaining three patients had a R_1_-delta of 0.007, 0.009 and 0.150.

When comparing hemispheres within patients, R_1_ was lower in the hemisphere ipsilateral to the ICA occlusion than in the contralateral hemisphere (Table 2; R_1_ ipsilateral 0.899 [IQR 0.876–0.921] versus contralateral 0.935 [IQR 0.889–0.970]). In the regional analyses, R_1_ was lower on the side ipsilateral to the occlusion than on the contralateral side in the frontal lobe, the parietal lobe, the paracentral gyrus, the precuneus and the insula (Table 2). There were no significant differences between hemispheres in the remaining regions.

**Table 2.** Hemispheric differences in cerebral perfusion and cerebral amyloid-β burden.

	Side ipsilateral to the ICA occlusion N = 20	Side contralateral to the ICA occlusion N = 20	p
*Cerebral perfusion, R_1_*			
*Entire hemisphere*	0.899 (0.876–0.921)	0.935 (0.889–0.970)	0.002*
*Lobe*			
Frontal lobe	0.924 (0.885–0.975)	0.955 (0.920–1.002)	0.002*
Temporal lobe	0.830 (0.806–0.853)	0.853 (0.825–0.890)	0.070
Parietal lobe	0.896 (0.854–0.933)	0.938 (0.875–0.971)	0.002*
Occipital lobe	0.964 (0.928–1.014)	0.975 (0.934–1.023)	0.033
*Alzheimer's region*			
Anterior cingulate	0.902 (0.858–0.955)	0.942 (0.887–0.968)	0.076
Posterior cingulate	1.094 (1.043–1.155)	1.102 (1.025–1.162)	0.388
Paracentral gyrus	0.866 (0.798–0.915)	0.907 (0.868–0.947)	0.001*
Lateral orbitofrontal cortex	0.985 (0.953–1.019)	0.995 (0.955–1.033)	0.648
Medial orbitofrontal cortex	0.935 (0.897–0.959)	0.939 (0.902–0.980)	0.452
Precuneus	0.907 (0.869–0.968)	0.958 (0.884–1.003)	0.011*
Insula	0.872 (0.841–0.895)	0.914 (0.878–0.951)	0.002*
*Watershed areas*	0.806 (0.738–0.847)	0.764 (0.710–0.844)	0.165
*Cerebral amyloid-β burden, BP_ND_*			
*Entire hemisphere*	0.172 (0.124–0.181)	0.168 (0.137–0.191)	0.083
*Lobe*			
Frontal lobe	0.188 (0.146–0.203)	0.185 (0.154–0.202)	0.368
Temporal lobe	0.152 (0.103–0.168)	0.149 (0.124–0.171)	0.571
Parietal lobe	0.157 (0.120–0.181)	0.158 (0.133–0.178)	0.105
Occipital lobe	0.175 (0.122–0.184)	0.178 (0.141–0.190)	0.246
*Alzheimer's region*			
Anterior cingulate	0.131 (0.109–0.185)	0.154 (0.117–0.188)	0.870
Posterior cingulate	0.158 (0.118–0.181)	0.141 (0.104–0.164)	0.202
Paracentral gyrus	0.171 (0.140–0.189)	0.175 (0.149–0.191)	0.027
Lateral orbitofrontal cortex	0.178 (0.153–0.259)	0.192 (0.153–0.216)	0.165
Medial orbitofrontal cortex	0.180 (0.141–0.198)	0.178 (0.135–0.205)	0.349
Precuneus	0.158 (0.129–0.182)	0.159 (0.132–0.187)	0.076
Insula	0.164 (0.140–0.185)	0.172 (0.146–0.206)	0.409
*Watershed areas*	0.163 (0.142–0.195)	0.167 (0.138–0.198)	0.246

Data are presented as median (interquartile range).

BP_ND_: binding potential; ICA: internal carotid artery; R_1_: relative perfusion.

*p < 0.05 after false discovery rate correction.

### Cerebral amyloid-β burden

Only one patient was amyloid-positive based on visual reading of the PET-scan. The median global BP_ND_ for all patients was 0.172 (IQR 0.129–0.187). Nine of 20 patients had a BP_ND_-delta of more than 0, i.e., higher ipsilateral cerebral Aβ burden (median BP_ND_-delta of −0.010 [IQR −0.015–0.006]).

BP_ND_ did not differ significantly between hemispheres (Table 2; BP_ND_ ipsilateral 0.172 [IQR 0.124–0.181] versus contralateral 0.168 [IQR 0.137–0.191]). Furthermore, there were no significant differences in BP_ND_ in the individual lobes, early Alzheimer's regions or watershed areas. Repeating the analyses in 17 patients with ipsilateral hypoperfusion, provided similar results (Supplemental Table 1).

### Association between cerebral perfusion and cerebral amyloid-β burden

**Figure 1.**
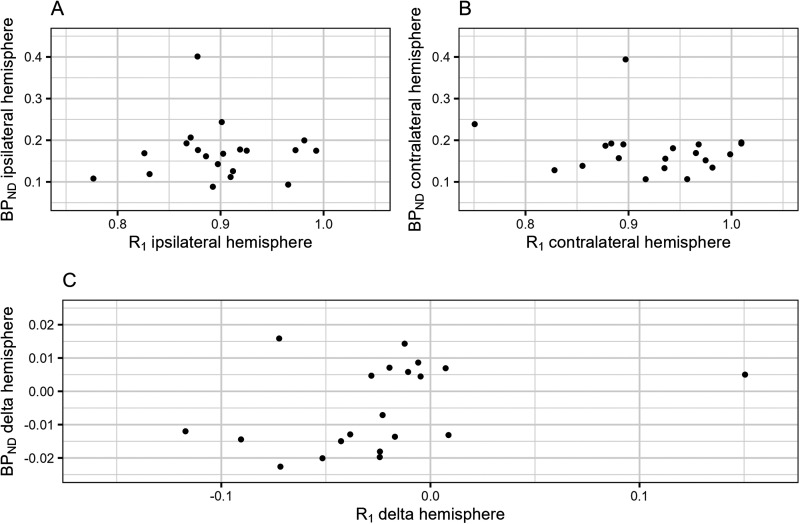
Associations between cerebral perfusion and cerebral amyloid-β burden. This figure shows the crude associations between R_1_ and BP_ND_ (A) within the hemisphere ipsilateral to the internal carotid artery occlusion, (B) within the hemisphere contralateral to the internal carotid artery occlusion and (C) between hemispheres. BP_ND_: binding potential; R_1_: relative perfusion.

There were no statistically significant associations between R_1_ and BP_ND_ within hemispheres, nor between the R_1_-delta and BP_ND_-delta (Figure 1, Supplemental Table 2).

### Potential determinants of R_1_-delta and BP_ND_-delta

**Figure 2.**
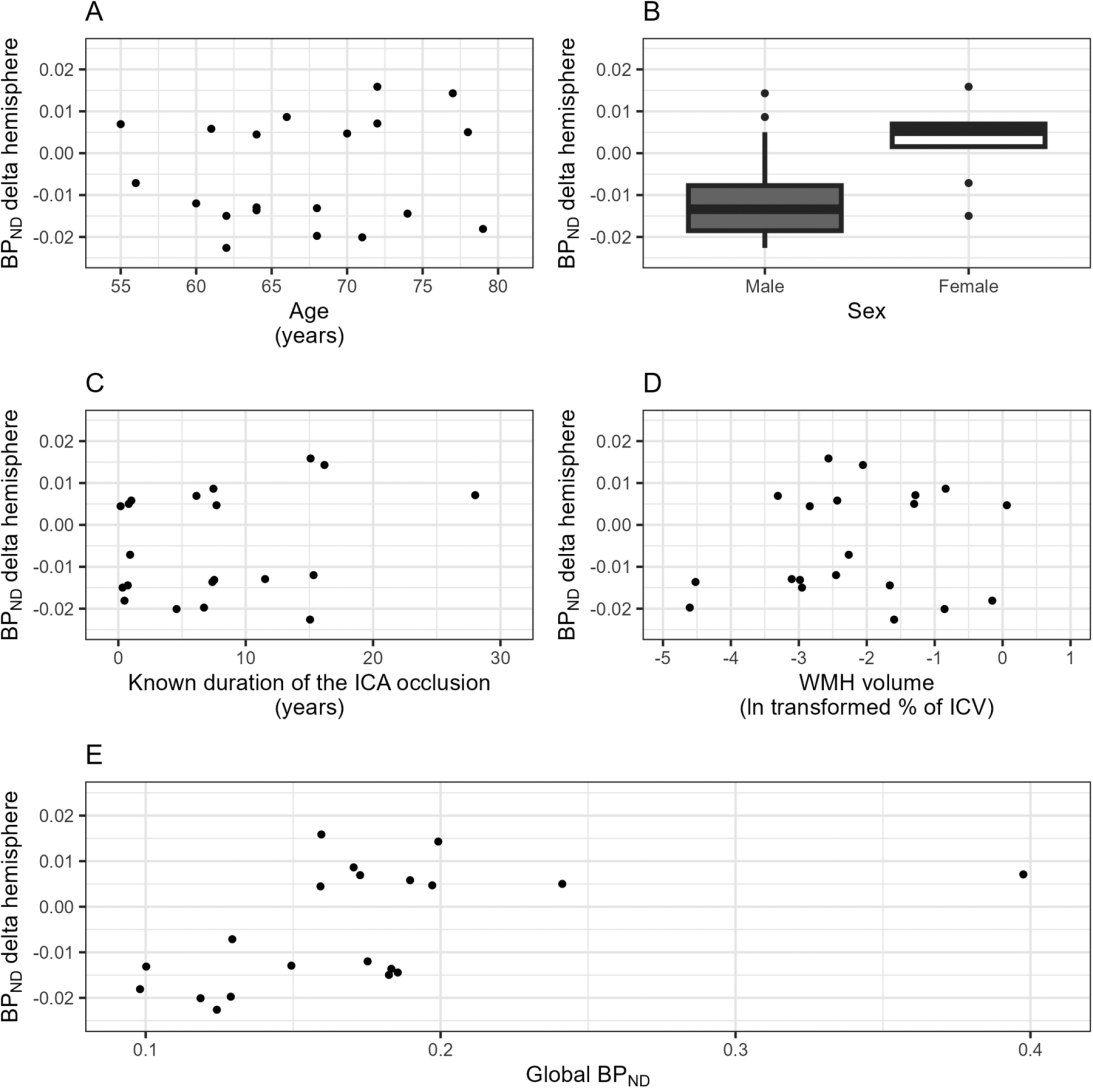
Associations between potential effect modifiers and the BP_ND_-delta. This figure shows the crude associations between the BP_ND_-delta and (A) age, (B) sex, (C) known duration of the ICA occlusion, (D) WMH volume, and (E) global BP_ND_. BP_ND_: binding potential; ICA: internal carotid artery; ICV: intracranial volume; IQR: interquartile range; WMH: white matter hyperintensity.

Age, sex, known duration of the occlusion, WMH volume and global R_1_ were not associated with the R_1_-delta (Supplemental Figure 1, Supplemental Table 3). Age, known duration of the occlusion, WMH volume and global BP_ND_ were not associated with the BP_ND_-delta (Figure 2, Supplemental Table 3). Female sex was associated with a significantly higher BP_ND_-delta than male sex after adjustment for confounders. Males tended to have more amyloid binding in the contralateral hemisphere, resulting in a negative BP_ND_-delta, whereas there was almost no interhemispheric difference in amyloid binding in females, resulting in a BP_ND_-delta of nearly zero.

### Cognitive functioning

Two participants had minor cognitive impairment (1 domain impaired) and 5 participants had major cognitive impairment (2 or more domains impaired). The domains attention-psychomotor speed (35%) and memory (20%) were most commonly affected, followed by executive functioning (10%) and language (5%).

Global R_1_ was not associated with global cognitive functioning (adjusted β 0.10, 95% CI −0.28–0.49), nor was global BP_ND_ (adjusted β −0.14, 95% CI −0.52–0.24) (Table 3). There were also no significant associations between global R_1_ or BP_ND_ and any of the specific cognitive domains.

**Table 3.** Associations between cerebral perfusion, cerebral amyloid-β burden and cognitive functioning.

	Unadjusted β (95% CI)	p	Adjusted β (95% CI)^a^	p
*Cerebral perfusion*				
Global cognitive functioning	0.29 (−0.06–0.64)	0.098	0.10 (−0.28–0.49)	0.568
Memory	0.33 (−0.21–0.87)	0.218	0.11 (−0.52–0.74)	0.718
Language	0.28 (−0.08–0.63)	0.116	0.16 (−0.26–0.59)	0.423
Attention-psychomotor speed	0.24 (−0.36–0.85)	0.412	−0.04 (−0.71–0.64)	0.907
Executive functioning	0.30 (0.04–0.57)	0.027	0.18 (−0.08–0.44)	0.153
*Cerebral amyloid-β burden*				
Global cognitive functioning	−0.11 (−0.48–0.26)	0.547	−0.14 (−0.52–0.24)	0.437
Memory	0.20 (−0.36–0.76)	0.459	0.08 (−0.55–0.71)	0.779
Language	−0.08 (−0.46–0.30)	0.667	−0.11 (−0.54–0.32)	0.600
Attention-psychomotor speed	−0.32 (−0.91–0.28)	0.279	−0.35 (−1.00–0.31)	0.275
Executive functioning	−0.24 (−0.52–0.04)	0.088	−0.20 (−0.45–0.06)	0.123

Cerebral perfusion is modeled per 1 standard deviation increase in relative perfusion (R_1_). Cerebral amyloid-β burden is modeled per 1 standard deviation increase in binding potential (BP_ND_). None of the associations remained statistically significant after false discovery rate correction.

^a^ Adjusted for age, sex, and years of education.

After exclusion of the visually amyloid-positive patient, findings remained essentially unchanged (Supplemental Table 4). For executive functioning specifically, the association attenuated towards zero. Assessing lobar R_1_ in relation to cognitive functioning, revealed that higher R_1_ in the temporal lobe and occipital lobe related to a better global cognitive function, driven by differences in memory and language for temporal lobe R_1_ and by differences in language and executive functioning for occipital lobe R_1_ (Supplemental Table 5). These associations attenuated after adjustment for confounders and became not statistically significant. Lobar BP_ND_ was not associated with cognitive functioning (Supplemental Table 6). There was no significant interaction between age or sex and R_1_ or BP_ND_ in relation to cognitive functioning, nor was there an interaction between R_1_ and BP_ND_ (FDR-corrected p-interaction all > 0.05).

## Discussion

We found lower cerebral perfusion in the hemisphere ipsilateral to the occlusion than in the contralateral hemisphere in our specifically selected patients with a unilateral, complete ICA occlusion. However, this did not result in an asymmetrical cerebral Aβ burden. Furthermore, neither cerebral perfusion nor Aβ burden were associated with cognitive functioning.

We reasoned that the first step towards cognitive impairment in patients with a unilateral ICA occlusion could be reduced ipsilateral cerebral perfusion. While it is generally accepted that patients with a unilateral ICA occlusion are hemodynamically impaired, it has been questioned repeatedly whether this hemodynamic impairment is severe enough to result in ipsilateral cerebral hypoperfusion. Indeed, a lower cerebral perfusion in the ipsilateral hemisphere has been found in some studies,^4–6^ but not in other previous studies.^26–28^ Regional differences in cerebral perfusion have been described with larger asymmetries in regions that are primarily dependent on flow from the ICA and MCA, namely the frontal and parietal regions.^5,6^ In the current study, we have shown that the hemodynamic impairment due to the ICA occlusion resulted in cerebral perfusion asymmetry between hemispheres, predominantly in the frontal and parietal lobes, thereby proving that our model of unilateral hypoperfusion is valid.

After establishing ipsilateral cerebral hypoperfusion, we then investigated whether this might be accompanied by a higher burden of Aβ in the ipsilateral hemisphere. With the exception of one patient, we did not find substantial Aβ binding at all. There were no differences in Aβ binding between hemispheres, with notably also no differences between hemispheres in the subgroup of patients with unilateral hypoperfusion. Previous studies examining cerebral Aβ burden in participants with or without a stenosis of the ICA also did not find a significant difference in cerebral Aβ burden on PET^29^ or postmortem neuropathological examination between patients and controls.^30^ Similarly, none of the studies, although few and with a small sample size, assessing differences within patients with a unilateral stenosis or occlusion of the ICA or MCA, found a significant difference in Aβ between the ipsilateral and contralateral hemisphere.^7,9–12^ However, only two of those studies used dynamic PET, as we did, to ensure that limited radiotracer uptake was not caused by impaired radiotracer delivery because of the COD, thereby accurately quantifying the Aβ load.^14^

We did not find evidence that cerebral hypoperfusion was associated with increased cerebral Aβ burden in patients with a unilateral ICA occlusion. Yet, given that our study population overall had low levels of Aβ, it might be more difficult to find an association between cerebral hypoperfusion and Aβ. However, we had expected to already find some Aβ pathology, even in the absence of clear clinical manifestation of cognitive decline, in line with the slow accumulation of Alzheimer pathology and long preclinical stage.^31^

Although 35% of the patients had at least one impaired cognitive domain, which is comparable in both frequency and cognitive profile to a previous cohort of patients with COD who underwent a similar neuropsychological assessment,^32,33^ variation in cognitive performance remained limited in this sample of 20 patients. This probably reduced our sensitivity to detect significant associations with cognitive functioning. Previous studies were able to link decreased cerebral perfusion to a reduced cognitive functioning in COD patients,^34,35^ though these findings were not confirmed in other studies.^33,36^ Moreover, preclinical animal studies reported that animals with both carotid occlusion and Aβ performed worse on a spatial memory task than animals with carotid occlusion only.^37–40^ Although the association between Aβ and cognitive functioning has not been examined in patients with carotid occlusion specifically, the association between Aβ and cognitive functioning in community-based cohorts is well established.^41,42^ Alternatively, cerebral perfusion or Aβ accumulation may not fully explain the frequently observed cognitive impairment in patients with COD. Instead, the hemodynamic vulnerability of COD patients may need to result in structural vascular brain injury, such as WMH or ischemic stroke, in order to affect cognitive functioning. Indeed, chronic white matter damage, particularly in several strategic white matter tracts, relates to worse cognitive performance in COD patients.^43^ COD is a known risk factor for cognitive impairment after stroke.^44^ Non-vascular mechanisms, such as inflammation, may also form the link between atherosclerosis and Alzheimer's disease.^45^

The main strength of our study is the detailed phenotyping using a standardized neuropsychological assessment in combination with the dynamic PET-scanning. This enabled us to measure and verify unilateral cerebral hypoperfusion and correct for that in the quantification of the Aβ load. Our cohort of patients with longstanding, complete unilateral ICA occlusions is relatively large compared to previous studies that have made within patient comparisons of Aβ burden in patients with COD. Although the still limited size of the cohort renders our study susceptible to type II errors and may also give a single outlier a substantial influence on the results. The specific characteristics of our cohort make them suitable to examine our hypothesis, but those characteristics also bring limitations regarding the generalizability. As by design, we excluded patients with a contralateral stenosis or history of vascular reconstructive surgery, which are frequent characteristics of patients with a ICA occlusion. Moreover, the fact that the patients performed well in daily living despite their ICA occlusion, may have limited our chances of finding higher levels of cerebral Aβ. Arguments can be made that the overall Aβ burden was too low to detect any differences between hemispheres or any associations with cerebral perfusion or cognitive functioning (i.e., floor effect). Similar reasoning can be applied to the limited amount of variation in cognitive functioning, which also hampered our ability to find significant associations with either cerebral perfusion or cerebral Aβ. Furthermore, R_1_ is not a direct measure of cerebral blood flow, but rather the influx (i.e., flow multiplied by extraction) into the target region relative to the influx into the reference region, in our case the cerebellar gray matter. Yet it is generally assumed that the extraction of various amyloid tracers is constant in different brain regions, which makes R_1_ a marker for relative cerebral blood flow.^46^ Finally, performing multiple analyses increases the risk of type I errors, which we tried to mitigate by applying FDR-correction.

The complex pathway leading to cognitive impairment in patients with COD remains to be fully elucidated. This study unfortunately did not find a target for treatment in cerebral perfusion or Aβ burden, leaving current treatment options limited to managing traditional vascular risk factors, such as hypertension. Cerebral Aβ in particular, could have made a promising target, in light of the recent developments in anti-amyloid treatment. Future research may instead focus on studying inflammation as a potential contributor to cognitive decline.

In conclusion, patients with a unilateral ICA occlusion have a lower cerebral perfusion in the hemisphere ipsilateral to the occlusion than in the contralateral hemisphere. However, this was not accompanied by an asymmetrical cerebral Aβ burden. Furthermore, neither cerebral perfusion nor Aβ burden were associated with cognitive functioning. Our findings indicate that cognitive impairment in patients with ICA occlusion is not due to exacerbated accumulation of Aβ in the brain.

## Supplemental Material

sj-docx-1-alz-10.1177_13872877251329593 - Supplemental material for Dynamic PET imaging in patients with unilateral carotid occlusion shows lateralized cerebral hypoperfusion, but no amyloid binding Supplemental material, sj-docx-1-alz-10.1177_13872877251329593 for Dynamic PET imaging in patients with unilateral carotid occlusion shows lateralized cerebral hypoperfusion, but no amyloid binding by Naomi LP Starmans, Anna E Leeuwis, Edwin Bennink, Sebastiaan L Meyer Viol, Sandeep SV Golla, Jan Willem Dankbaar, Esther E Bron, Geert Jan Biessels, L Jaap Kappelle, Wiesje M van der Flier, Nelleke Tolboom and in Journal of Alzheimer's Disease
