# Supplementary material for: Dynamic PET imaging in patients with unilateral carotid occlusion shows lateralized cerebral hypoperfusion, but no amyloid binding
Source: J Alzheimers Dis. 2025 Apr 17;105(2):519–30. doi: 10.1177/13872877251329593 (PMC12231833; doi:10.1177/13872877251329593)
Supplement: sj-docx-1-alz-10.1177_13872877251329593 - Supplemental material for Dynamic PET imaging in patients with unilateral carotid occlusion shows lateralized cerebral hypoperfusion, but no amyloid binding [file sj-docx-1-alz-10.1177_13872877251329593.docx]

**Supplemental Material**

**Dynamic PET imaging in patients with unilateral carotid occlusion shows lateralized cerebral hypoperfusion, but no amyloid binding**

**Supplemental Table 1.** Hemispheric differences in cerebral amyloid-β burden in patients with ipsilateral hypoperfusion

|  | **Side ipsilateral to**  **the ICA occlusion**  **N = 17** | **Side contralateral to**  **the ICA occlusion**  **N = 17** | **p** |
| --- | --- | --- | --- |
| **Cerebral amyloid-β burden, BP_ND_** |  |  |  |
| *Entire hemisphere* | 0.169 (0.126 – 0.178) | 0.166 (0.138 – 0.190) | 0.080 |
| *Lobe* |  |  |  |
| Frontal lobe | 0.185 (0.150 – 0.199) | 0.184 (0.155 – 0.201) | 0.579 |
| Temporal lobe | 0.152 (0.106 – 0.168) | 0.150 (0.124 – 0.180) | 0.306 |
| Parietal lobe | 0.156 (0.123 – 0.179) | 0.155 (0.134 – 0.175) | 0.109 |
| Occipital lobe | 0.174 (0.123 – 0.181) | 0.178 (0.141 – 0.190) | 0.098 |
| *Alzheimer’s region* |  |  |  |
| Anterior cingulate | 0.124 (0.107 – 0.168) | 0.154 (0.118 – 0.187) | 0.174 |
| Posterior cingulate | 0.158 (0.119 – 0.180) | 0.143 (0.114 – 0.158) | 0.244 |
| Paracentral gyrus | 0.167 (0.149 – 0.188) | 0.170 (0.153 – 0.186) | 0.064 |
| Lateral orbitofrontal cortex | 0.179 (0.155 – 0.258) | 0.195 (0.159 – 0.215) | 0.089 |
| Medial orbitofrontal cortex | 0.187 (0.146 – 0.198) | 0.185 (0.138 – 0.213) | 0.712 |
| Precuneus | 0.158 (0.132 – 0.181) | 0.152 (0.133 – 0.185) | 0.109 |
| Insula | 0.166 (0.141 – 0.181) | 0.169 (0.148 – 0.205) | 1.000 |
| *Watershed areas* | 0.163 (0.142 – 0.189) | 0.163 (0.139 – 0.198) | 0.355 |

Data are presented as median (interquartile range). Differences were already not statistically significant before false discovery rate correction.

BP_ND_: binding potential; ICA: internal carotid artery.

**Supplemental Table 2.** Associations between cerebral perfusion and cerebral amyloid-β burden

|  | **Unadjusted β**  **(95% CI)** | **p** | **Adjusted β**  **(95% CI)^a^** | **p** |
| --- | --- | --- | --- | --- |
| **Within hemispheres** |  |  |  |  |
| Ipsilateral R_1_ ~ ipsilateral BP_ND_ | 0.05 (-0.58 – 0.67) | 0.880 | -0.05 (-0.64 – 0.54) | 0.858 |
| Contralateral R_1_ ~ contralateral BP_ND_ | -0.17 (-0.62 – 0.28) | 0.443 | -0.17 (-0.63 – 0.30) | 0.457 |
| **Between hemispheres** |  |  |  |  |
| R_1_ delta ~ BP_ND_ delta | 0.08 (-0.03 – 0.20) | 0.130 | 0.07 (-0.03 – 0.17) | 0.170 |

BP_ND_: binding potential, R_1_: relative perfusion. Associations were already not statistically significant before false discovery rate correction.

^a^Adjusted for age and sex.

**Supplemental Table 3.** Associations between potential effect modifiers and the R_1_-delta or the BP_ND_-delta

|  | **Unadjusted β**  **(95% CI)** | **p** | **Adjusted β**  **(95% CI)^a^** | **p** |
| --- | --- | --- | --- | --- |
| **Cerebral perfusion,**  **R_1_ delta** |  |  |  |  |
| Age, y | 0.002 (-0.002 – 0.006) | 0.293 | 0.002 (-0.002 – 0.006) | 0.266 |
| Female sex | 0.000 (-0.052 – 0.052) | 0.992 | 0.012 (-0.044 – 0.068) | 0.661 |
| Known duration of the occlusion, y | -0.002 (-0.005 – 0.001) | 0.223 | -0.002 (-0.006 – 0.001) | 0.191 |
| WMH volume, ln transformed % of ICV | 0.002 (-0.019 – 0.022) | 0.863 | -0.005 (-0.030 – 0.019) | 0.646 |
| Global cerebral perfusion, R_1_ | -0.241 (-0.710 – 0.228) | 0.295 | -0.215 (-0.727 – 0.297) | 0.387 |
| **Cerebral amyloid-β burden, BP_ND_ delta** |  |  |  |  |
| Age, y | 0.000 (-0.001 – 0.001) | 0.616 | 0.001 (-0.000 – 0.001) | 0.099 |
| Female sex | 0.013 (0.002 – 0.024) | 0.023 | 0.016 (0.005 – 0.027) | 0.007* |
| Known duration of the occlusion, y | 0.000 (-0.000 – 0.001) | 0.303 | 0.000 (-0.000 – 0.001) | 0.251 |
| WMH volume, ln transformed % of ICV | 0.002 (-0.003 – 0.007) | 0.493 | -0.000 (-0.005 – 0.005) | 0.977 |
| Global cerebral amyloid-β burden, BP_ND_ | 0.099 (0.014 – 0.184) | 0.024 | 0.056 (-0.034 – 0.146) | 0.208 |

BP_ND_: binding potential, ICV: intracranial volume, ln: natural log, R_1_: relative perfusion, WMH: white matter hyperintensities.

^a^Adjusted for age and sex.

*p < 0.05 after false discovery rate correction.

**Supplemental Table 4.** Associations between cerebral amyloid-β burden and cognitive functioning in amyloid-negative patients

|  | **Unadjusted β**  **(95% CI)** | **p** | **Adjusted β**  **(95% CI)^a^** | **p** |
| --- | --- | --- | --- | --- |
| **Cerebral amyloid-β burden** |  |  |  |  |
| Global cognitive functioning | -0.11 (-0.79 – 0.57) | 0.727 | -0.33 (-0.98 – 0.33) | 0.306 |
| Memory | 0.17 (-0.84 – 1.19) | 0.722 | -0.15 (-1.26 – 0.95) | 0.770 |
| Language | -0.35 (-1.02 – 0.32) | 0.283 | -0.54 (-1.24 – 0.16) | 0.121 |
| Attention-psychomotor speed | -0.36 (-1.45 – 0.73) | 0.494 | -0.62 (-1.76 – 0.52) | 0.266 |
| Executive functioning | 0.08 (-0.39 – 0.55) | 0.723 | 0.00 (-0.43 – 0.44) | 0.982 |

Cerebral amyloid-β burden is modeled per 1 standard deviation increase in binding potential (BP_ND_). Associations were already not statistically significant before false discovery rate correction.

^a^Adjusted for age, sex, and years of education.

**Supplemental Table 5.** Associations between lobar perfusion and cognitive functioning

|  | **Unadjusted β**  **(95% CI)** | **p** | **Adjusted β**  **(95% CI)^a^** | **p** |
| --- | --- | --- | --- | --- |
| **Frontal lobe** |  |  |  |  |
| Global cognitive function | -0.06 (-0.43 – 0.32) | 0.758 | -0.24 (-0.60 – 0.11) | 0.160 |
| Memory | -0.11 (-0.67 – 0.46) | 0.693 | -0.42 (-0.99 – 0.15) | 0.140 |
| Language | -0.03 (-0.41 – 0.35) | 0.881 | -0.16 (-0.58 – 0.26) | 0.424 |
| Attention-psychomotor speed | -0.22 (-0.83 – 0.39) | 0.458 | -0.47 (-1.08 – 0.15) | 0.125 |
| Executive functioning | 0.13 (-0.17 – 0.43) | 0.364 | 0.07 (-0.20 – 0.34) | 0.599 |
| **Temporal lobe** |  |  |  |  |
| Global cognitive function | 0.48 (0.18 – 0.77) | 0.003* | 0.36 (0.03 – 0.68) | 0.032 |
| Memory | 0.62 (0.14 – 1.10) | 0.014* | 0.52 (-0.02 – 1.07) | 0.060 |
| Language | 0.52 (0.24 – 0.80) | 0.001* | 0.46 (0.12 – 0.80) | 0.011 |
| Attention-psychomotor speed | 0.48 (-0.09 – 1.05) | 0.096 | 0.27 (-0.38 – 0.91) | 0.391 |
| Executive functioning | 0.29 (0.02 – 0.56) | 0.037 | 0.17 (-0.08 – 0.43) | 0.170 |
| **Parietal lobe** |  |  |  |  |
| Global cognitive function | 0.32 (-0.02 – 0.66) | 0.067 | 0.12 (-0.26 – 0.51) | 0.510 |
| Memory | 0.33 (-0.21 – 0.88) | 0.213 | 0.11 (-0.53 – 0.75) | 0.727 |
| Language | 0.24 (-0.12 – 0.60) | 0.175 | 0.10 (-0.34 – 0.54) | 0.637 |
| Attention-psychomotor speed | 0.37 (-0.22 – 0.96) | 0.205 | 0.09 (-0.60 – 0.78) | 0.790 |
| Executive functioning | 0.32 (0.07 – 0.58) | 0.017* | 0.20 (-0.07 – 0.46) | 0.131 |
| **Occipital lobe** |  |  |  |  |
| Global cognitive function | 0.45 (0.14 – 0.75) | 0.006* | 0.34 (0.02 – 0.65) | 0.038 |
| Memory | 0.55 (0.05 – 1.05) | 0.032 | 0.47 (-0.07 – 1.01) | 0.085 |
| Language | 0.43 (0.12 – 0.75) | 0.009* | 0.38 (0.02 – 0.73) | 0.040 |
| Attention-psychomotor speed | 0.43 (-0.15 – 1.01) | 0.139 | 0.24 (-0.39 – 0.87) | 0.423 |
| Executive functioning | 0.38 (0.14 – 0.62) | 0.004* | 0.26 (0.04 – 0.48) | 0.024 |

Lobar perfusion is modeled per 1 standard deviation increase in lobar relative perfusion (R_1_).

^a^Adjusted for age, sex, and years of education.

*p < 0.05 after false discovery rate correction.

**Supplemental Table 6.** Associations between lobar amyloid-β burden and cognitive functioning

|  | **Unadjusted β**  **(95% CI)** | **p** | **Adjusted β**  **(95% CI)^a^** | **p** |
| --- | --- | --- | --- | --- |
| **Frontal lobe** |  |  |  |  |
| Global cognitive function | -0.17 (-0.54 – 0.19) | 0.330 | -0.20 (-0.56 – 0.17) | 0.270 |
| Memory | 0.12 (-0.44 – 0.68) | 0.661 | 0.01 (-0.61 – 0.64) | 0.969 |
| Language | -0.13 (-0.50 – 0.25) | 0.488 | -0.15 (-0.57 – 0.28) | 0.473 |
| Attention-psychomotor speed | -0.41 (-1.00 – 0.17) | 0.152 | -0.43 (-1.06 – 0.20) | 0.168 |
| Executive functioning | -0.28 (-0.55 – -0.01) | 0.046 | -0.23 (-0.47 – 0.02) | 0.069 |
| **Temporal lobe** |  |  |  |  |
| Global cognitive function | 0.03 (-0.34 – 0.41) | 0.863 | -0.04 (-0.43 – 0.34) | 0.821 |
| Memory | 0.33 (-0.22 – 0.87) | 0.225 | 0.17 (-0.45 – 0.80) | 0.561 |
| Language | 0.07 (-0.31 – 0.45) | 0.716 | 0.02 (-0.42 – 0.45) | 0.937 |
| Attention-psychomotor speed | -0.11 (-0.72 – 0.51) | 0.719 | -0.19 (-0.86 – 0.47) | 0.545 |
| Executive functioning | -0.16 (-0.45 – 0.13) | 0.271 | -0.16 (-0.42 – 0.10) | 0.210 |
| **Parietal lobe** |  |  |  |  |
| Global cognitive function | -0.09 (-0.46 – 0.29) | 0.628 | -0.12 (-0.50 – 0.26) | 0.503 |
| Memory | 0.27 (-0.28 – 0.82) | 0.309 | 0.17 (-0.45 – 0.79) | 0.566 |
| Language | -0.05 (-0.43 – 0.32) | 0.765 | -0.08 (-0.51 – 0.35) | 0.689 |
| Attention-psychomotor speed | -0.32 (-0.92 – 0.27) | 0.272 | -0.36 (-1.01 – 0.29) | 0.252 |
| Executive functioning | -0.25 (-0.53 – 0.03) | 0.077 | -0.22 (-0.47 – 0.04) | 0.087 |
| **Occipital lobe** |  |  |  |  |
| Global cognitive function | -0.05 (-0.42 – 0.33) | 0.785 | -0.10 (-0.49 – 0.29) | 0.601 |
| Memory | 0.25 (-0.31 – 0.80) | 0.365 | 0.11 (-0.53 – 0.74) | 0.731 |
| Language | -0.11 (-0.48 – 0.27) | 0.565 | -0.17 (-0.60 – 0.26) | 0.415 |
| Attention-psychomotor speed | -0.25 (-0.85 – 0.36) | 0.398 | -0.30 (-0.97 – 0.36) | 0.347 |
| Executive functioning | -0.09 (-0.39 – 0.21) | 0.546 | -0.02 (-0.30 – 0.26) | 0.878 |

Lobar amyloid-β burden is modeled per 1 standard deviation increase in lobar binding potential (BP_ND_).

^a^Adjusted for age, sex, and years of education.

*p < 0.05 after false discovery rate correction.


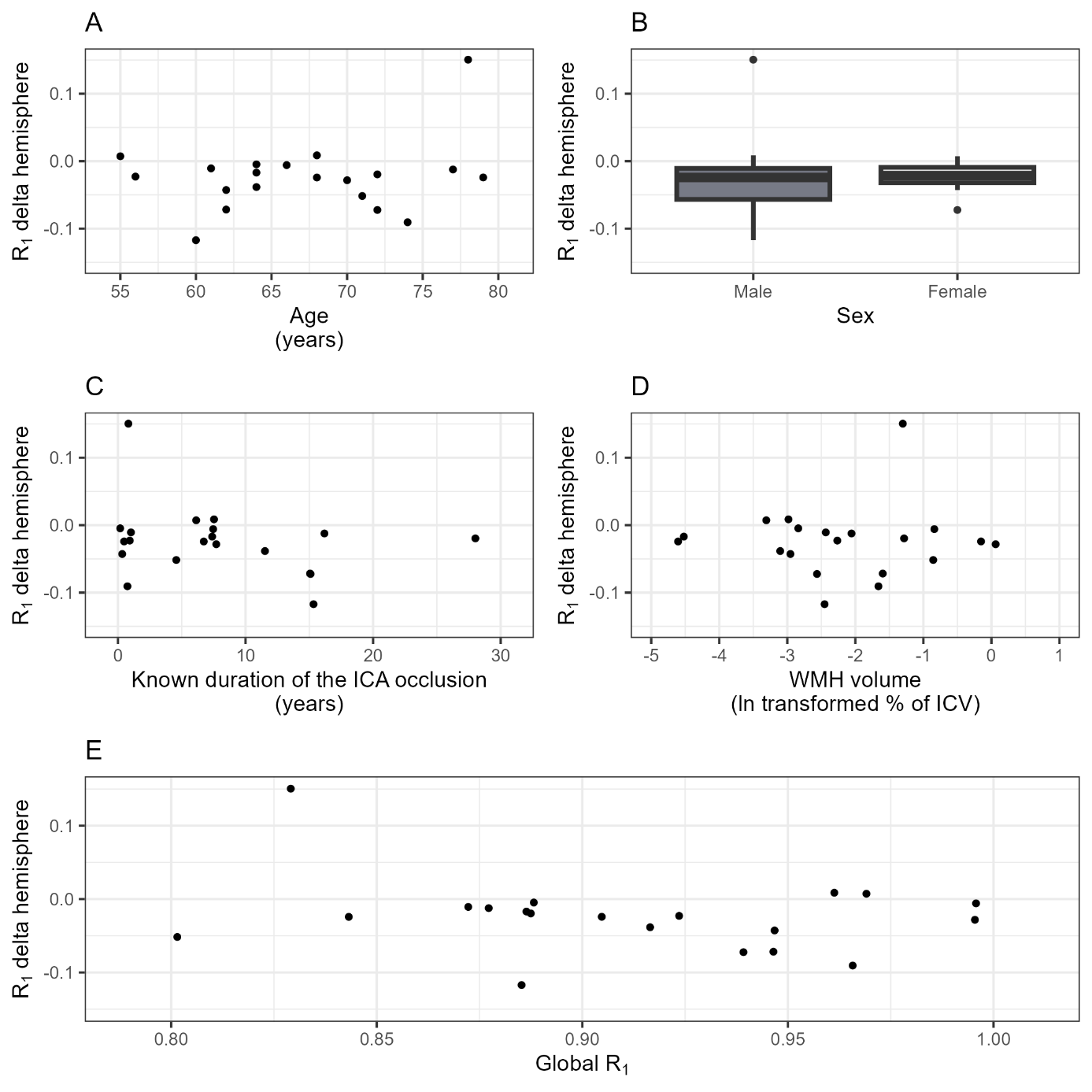


**Supplemental Figure 1.** Associations between potential effect modifiers and the R_1_-delta. This figure shows the crude associations between the R_1_-delta and (A) age, (B) sex, (C) known duration of the ICA occlusion, (D) WMH volume, and (E) global R_1_. ICA: internal carotid artery; ICV: intracranial volume; IQR: interquartile range; R_1_: relative perfusion; WMH: white matter hyperintensity**.**
